# Stroke to Dementia Associated with Environmental Risks—A Semi-Markov Model

**DOI:** 10.3390/ijerph17061944

**Published:** 2020-03-16

**Authors:** Kung-Jeng Wang, Chia-Min Lee, Gwo-Chi Hu, Kung-Min Wang

**Affiliations:** 1 Department of Industrial Management, National Taiwan University of Science and Technology, Taipei 106, Taiwan; m10501003@mail.ntust.edu.tw; 2 Department of Rehabilitation Medicine, Mackay Memorial Hospital, Number 92, Section 2, Zhongshan North Road, Zhongshan District, Taipei City 10449, Taiwan; kung527@yahoo.com.tw; 3 Department of Surgery, Shin-Kong Wu Ho-Su Memorial Hospital, Shilin District, Taipei 111, Taiwan; albert.hua@msa.hinet.net

**Keywords:** chronic disease, dementia, environmental risk, multivariate analysis, rehabilitation, stroke, semi-Markov process

## Abstract

Background: Most stroke cases lead to serious mental and physical disabilities, such as dementia and sensory impairment. Chronic diseases are contributory risk factors for stroke. However, few studies considered the transition behaviors of stroke to dementia associated with chronic diseases and environmental risks. Objective: This study aims to develop a prognosis model to address the issue of stroke transitioning to dementia associated with environmental risks. Design: This cohort study used the data from the National Health Insurance Research Database in Taiwan. Setting: Healthcare data were obtained from more than 25 million enrollees and covered over 99% of Taiwan’s entire population. Participants: In this study, 10,627 stroke patients diagnosed from 2000 to 2010 in Taiwan were surveyed. Methods: A Cox regression model and corresponding semi-Markov process were constructed to evaluate the influence of risk factors on stroke, corresponding dementia, and their transition behaviors. Main Outcome Measure: Relative risk and sojourn time were the main outcome measure. Results: Multivariate analysis showed that certain environmental risks, medication, and rehabilitation factors highly influenced the transition of stroke from a chronic disease to dementia. This study also highlighted the high-risk populations of stroke patients against the environmental risk factors; the males below 65 years old were the most sensitive population. Conclusion: Experiments showed that the proposed semi-Markovian model outperformed other benchmark diagnosis algorithms (i.e., linear regression, decision tree, random forest, and support vector machine), with a high *R*^2^ of 90%. The proposed model also facilitated an accurate prognosis on the transition time of stroke from chronic diseases to dementias against environmental risks and rehabilitation factors.

## 1. Introduction 

Stroke results from the sudden occlusion or rupture of a blood vessel that supplies blood to the brain. This condition is categorized as either ischemic or hemorrhagic, where 87% of such cases are ischemic [1]. Approximately 15 million people suffer from stroke each year, and the number of stroke deaths increases annually [2]. In Taiwan, stroke is the leading cause of death, which places a substantial burden on the national healthcare system [3]. Stroke is also one of the largest causes of serious long-term mental and physical disabilities [1,4,5] and a relevant factor in dementia development [6]. Previous studies proposed that poststroke dementia is a designation for dementia following stroke over time [7,8,9,10].

An estimated 12.6 million people died as a result of living or working in an unhealthy environment. This number is nearly one-fourth of the total global deaths. Among the top causes of environment-related deaths, stroke causes 2.5 million deaths annually [2]. In addition, chronic diseases, stroke, and dementia are closely related. Mortality and readmission of stroke patients are related to multiple chronic diseases [11]. Chronic hypertension, diabetes, and hyperlipidemia are important risk factors for stroke [3]. 

Stroke can be initially prevented by identifying risk factors. This condition is directly related to chronic diseases, specifically diabetes mellitus [12,13], hypertension [14], and hyperlipidemia [15]. 

In terms of environmental interfering factors, the trends in stroke rates are associated with changing exposures to population risk factors [16]. Considering the development of the country, its exposure to high levels of air pollutants related to combustion increases the risk of stroke in people [17,18]. Some studies proclaimed that seasonal variation and temperature trigger the incidence of stroke [19], and cold temperature is positively correlated with stroke admissions [20,21]. 

The risk interfering factors in stroke may also be related to socioeconomic factors, such as marital status [22], low socioeconomic status, and elderly living alone [23].

Dementia is a frequent outcome of stroke, which increases the probability of long-term disability and mortality [24]. The presumed etiology of poststroke dementia indicates that two-thirds of the patients have vascular dementia and one-third of those have Alzheimer’s disease [7,25,26,27].

Few studies considered the transition behavior in stroke to dementia, which is associated with chronic diseases, environmental risks, medication, and rehabilitation factors due to high complexity with multivariates and multilayers [28,29,30]. Therefore, the present study aims to address this issue. We developed a semi-Markovian process (SMP) to represent multiple states with time-based transition behavior of stroke to dementia. The objectives of this study were as follows: (i) to identify the environmental risk factors associated with stroke and (ii) to predict the transition of stroke from a chronic disease to dementia associated with environmental risks and rehabilitation via SMP.

## 2. Materials and Methods 

### 2.1. Population

**Figure 1:**
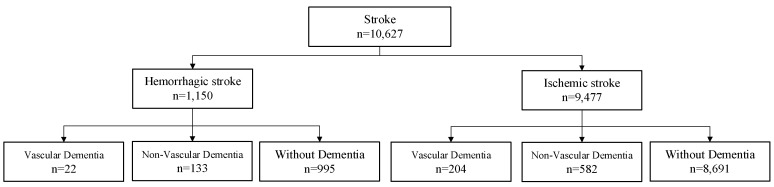
Stroke population in the study.

The population surveyed in this study was from National Health Insurance Research Database (NHIRD) in Taiwan and consisted of the detailed healthcare data from more than 25 million enrollees and covered over 99% of Taiwan’s entire population [31]. This article does not contain any studies with human participants or animals performed by any of the authors. The accuracy of stroke diagnosis in NHIRD has been validated in previous research [32]. A total of 10,627 stroke patients were selected, and the selection procedure is depicted in Figure 1. Stroke patients diagnosed with stroke between 2000 and 2010 during at least three consecutive clinic visits or hospitalization were screened. Patients younger than 30 years old and those diagnosed with hemorrhagic and ischemic stroke on the same day were excluded. This study did not explore the cases of stroke in youth because stroke often occurs at middle age. Furthermore, the rare case of simultaneous occurrence of the two types of stroke was excluded.

The criteria for enrollment in NHIRD included two types of stroke identified from January 1, 2000 to December 31, 2010. In International Classification of Diseases (ICD) ICD-9-CM coding system, stroke patients were defined by ICD-9-CM code of 430,432 for hemorrhagic stroke and ICD-9-CM code of 433,437 for ischemic stroke. The ICD-9-CM code for relative chronic diseases and dementia is listed in Table 1. For the modeling, the date of stroke diagnosis must be beyond the date of chronic disease diagnosis, and the date of dementia diagnosis must be beyond the date of stroke diagnosis.

Most of the stroke patients in Taiwan were diagnosed with hypertension, diabetes mellitus, and hyperlipidemia. This study used these three chronic diseases for the basic classification of stroke patients. Eleven types of chronic diseases were divided into 5 categories: newly diagnosed with hypertension, newly diagnosed with diabetes mellitus, newly diagnosed with hyperlipidemia, newly diagnosed with other chronic disease (including chronic lung disease, hyperthyroidism, chronic kidney disease, heart failure, atrial fibrillation, sleep apnea, gout, and peripheral artery disease), and stroke patients without any of the 11 chronic diseases before stroke. Furthermore, stroke patients usually suffer from multiple chronic diseases after being diagnosed with a chronic disease. This phenomenon is called the metabolic risk factor. Therefore, this study used the main types of chronic diseases including metabolic risk factor for re-classification. We used chi-square significance test for the selected chronic diseases and related metabolic risk factors to build an SMP model. 

### 2.2. Markovian-Based Modeling

Various approaches have been applied to explore the influence of key factors on stroke patients for occurrences over time; however, only a few achieved high prediction [33]. In addition to standard statistical analyses, such as correlation analysis, covariate analysis, and subgroup analysis, the transition probability in Markovian process is preferable to analyze the risk factor effect on stroke [33]. Among the variants, the SMP provides a flexible approach to characterize the distribution of the sojourn times between states. 

Multi-state models are often used to model the development of a disease in medical research, wherein the different levels of the disease can be seen as the states of the model [34]. The parametric SMP models allow the incorporation of covariates in the distribution of sojourn times to investigate the effect on the transition risk by using a proportional-hazard regression model [35,36].

The processes and the calculation of the transition probabilities of a SMP can be described as follows. First consider a model with *k* states belonging to finite state space *E* = {1, 2, …, *k*}, and consider *X*_0_, *X*_1_, *X*_2_, …, *X_n_* ∈ *E* be the successive states in *n* visits by a random process, in which 0 = *T*_0_ < *T*_1_ < … < *T_n_* are the consecutive time of entrance into each of these states. And the probability of the *n* transitions jumping from state *i* to *j*, embedded into model can be written as:

*P_ij_* = *P*(*X_n+_*_1_ = *j*|*X_n_* = *i*) (1)

As the Markov process does not deal with the sojourn time of the state transitions, the random process regards the transition sojourn time (*T_n_*_+1_ − *T_n_*) in an SMP and its distribution that satisfies:

(2) $$Q_{ij}(t)=P(X_{n+1}=j,T_{n+1}-T_{n}\leq t|X_{n}=i)$$

The probability density function of sojourn time in state *i* before passing to state *j* is given by:

(3) $$f_{ij}(t)=\underset{\Delta t\to 0}{\lim}\frac{P(t<T_{n+1}-T_{n}<t+\Delta t|X_{n+1}=j,X_{n}=i)}{\Delta t}$$

The cumulative probability function, *F_ij_*(*t*) and the corresponding survival function of waiting time in state *i*, *S_i_*(*t*) are defined by:

(4) $$F_{\mathrm{ij}}\left(t\right){=P(T}_{n+1}-{\;T}_{n}\leq t|X_{n+1}{=j,\;X}_{n}=i)=\frac{Q_{\mathrm{ij}}\left(t\right)}{P_{\mathrm{ij}}}$$

(5) $$S_{i}(t)=1-P(T_{n+1}-T_{n}\leq t|X_{n}=i)=\sum_{j\in E}P_{ij}(1-F_{ij}(t))$$

The hazard function of an SMP model, which represents the probability of transition towards state *j* between time *t* and *t* + ∆*t*, given that the process is in state *i* for a duration t can be drawn as follows:

(6) $$\begin{array}{c} \lambda_{ij}(t)=\underset{\Delta t\to 0}{\lim}\frac{P(X_{n+1}=j,t<T_{n+1}-T_{n}<t+\Delta t|X_{n}=i,T_{n+1}-T_{n}>t)}{\Delta t}\\ \;=\frac{P_{ij}f_{ij}(t)}{S_{i}(t)}=\frac{P_{ij}S_{ij}(t)\alpha_{ij}(t)}{S_{i}(t)},\;\begin{array}{c} i,j\in K\\ i\neq j \end{array}\\ \;\lambda_{ii}(t)=-\sum_{i\neq j}\lambda_{ij}(t) \end{array}$$

Let *m_ij_* be the covariate specific states transition *i* to *j* and the vector is represented by $z_{ij}=(z_{ij}^{1},z_{ij}^{2},\ldots ,z_{ij}^{m_{ij}})$, and the baseline hazard function of the transition *i* to *j* is $h_{0,ij}\left(x\right)$, the parameter vector in a Cox model is $\beta_{ij}^{\prime }$. The hazard function with covariates is defined by:

(7) $$h_{\mathrm{ij}}\left({x,z}_{\mathrm{ij}}\right){=h}_{0,\mathrm{ij}}\left(x\right)\cdot \exp(\beta_{\mathrm{ij}}^{\prime }\cdot z_{\mathrm{ij}})$$

Incorporating the Cox model can not only deal with multivariate models, but investigate the effect upon the relative risk (RR) of transferred stroke. Further, under the transition-specific strategy for covariates, applying Weibull distributions as the sojourn time distribution is more flexible to fit the hazard function, also generalizes the exponential distribution by two parameters *W*(*σ_ij_*, *v_ij_*), making the model well suit for various shapes, especially for medical research [35]. The Weibull function of the sojourn time distribution is defined by:

(8) $$\alpha_{ij}(t)=v_{ij}{\left(\frac{1}{\sigma_{ij}}\right)}^{v_{ij}}t^{v_{ij}-1},\forall t\geq 0,\forall \sigma_{ij}\geq 0,\forall v_{ij}\geq 0$$

where *σ_ij_*, *v_ij_* are scale and shape parameters.

Covariate analysis and Markovian-based prediction modeling were used in this study. The covariates included in the SMP model must be of categorical parameters as limitation (Table 2). The environmental variables are defined in Table 3. In this study, a value beyond one standard deviation of the mean was considered as high incidence group. 

The divorce and unemployment rates used in this study were from Socio-Economic Geographic Information System (SEGIS) [37]. The temperature data were from Taiwan Typhoon and Flood Research Institute (TTFRI) [38]. The air pollution index was from the environmental resource database of Environmental Protection Administration (EPA) [39]. The rate of elderly people living alone was from Ministry of Health and Welfare [40]. Each patient was characterized by one set of covariates to assess the influence of the environmental risk factors on the stroke patients. The connection between the individual patient and the environmental data was examined using the registered residence of the stroke patients recorded in the NHIRD. The environmental risk factors were collected from 22 administrative districts of Taiwan.

**Figure 2:**
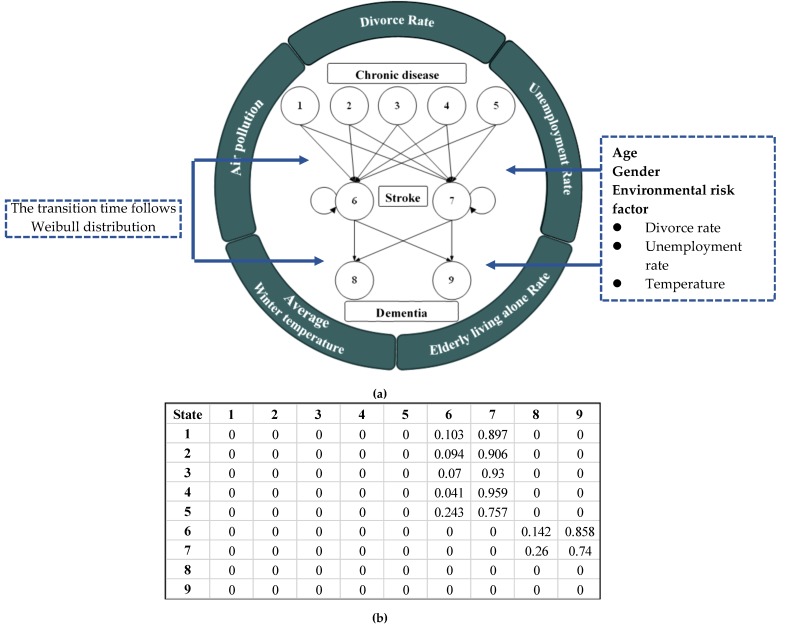
(**a**) Probability transition matrix; (**b**) The transition probability matrix in the SMP model.

The stroke patients were identified into nine states in the SMP model, including five states of chronic disease, two types of stroke, and two types of dementia, to explore the phenomenon among state transitions in SMP (Figure 2). This study combined hypertension and hyperlipidemia into one state to investigate the relationship between chronic disease and stroke. The state is defined in Table 4. Chronic disease states included hypertension and with one metabolic risk factor after diagnosed with hypertension, diabetes mellitus, hyperlipidemia and with one metabolic risk factor diagnosed with hyperlipidemia, with more than four metabolic risk factors after having another chronic disease, and without any of the 11 types of chronic disease. These chronic states represent the initial state in the SMP model. Hemorrhagic and ischemic stroke are the stroke states, whereas vascular dementia and non-vascular dementia are the dementia states.

The state diagram of the SMP model is depicted in Figure 2. The arrows indicate transitions among states, and the probability transition matrix was computed from our stroke patient population. This process used Weibull distribution to construct the time delay between different transitions. This study focused on the transferred stroke cases, such as chronic disease transfer to stroke state and stroke transfer to dementia state or remain in the stroke state. Such multi-state SMP model can be resolved via a software package called “semi-Markov” in R [33].

## 3. Results and Discussion

### 3.1. Multivariate Analysis 

In the covariate model, we obtained 98 transitions, consisting of 10 transitions in the first layer (5 types of chronic diseases multiplied by 2 types of stroke) and 4 transitions in the second layer (2 types of stroke multiplied by 2 types of dementia) for the 7 covariates (i.e., gender, age, and the five types of environmental risk factors). A Wald test (H0: βij = 0; H1: βij ≠ 0) with *p*-value < 0.05 was given. 

In the multivariate model, all covariates selected from the statistically significant transitions from univariate analysis were included. The results of multivariate analysis are presented in Table 5. The three sets of models were as follows: A. environmental risk factors, B. medication and rehabilitation factors, and C. all factors. The negative estimation β for the Cox regression model indicated that the transition risk was higher for the base group than for the other groups.

In the transition of chronic diseases to stroke, in model A, age, unemployment rate, and temperature showed high effect occurrences. The risk to transfer from hypertension (RR = 1.24) and hyperlipidemia (RR = 1.64) to hemorrhagic stroke was higher in males than in females as the base group. For the age factor, the patient under 65 years old has higher risk. The divorce rate indicated that high divorce rate was 3.91 times risk in diabetes mellitus transfer to hemorrhagic stroke group than in the base group. Conversely, the unemployment rate factor indicated that the base group had higher risk compared with the high unemployment rate. The temperature factor revealed that low temperature had 1.27–1.72 times risk compared with normal temperature. The air pollution revealed that high air pollution had 1.07 and 1.31 times risk to be transferred from hypertension and with other eight chronic diseases to hemorrhagic stroke.

In the transition of stroke to dementia, only the normal temperature (the base group) had high risk, such as in ischemic stroke transfer to vascular dementia (RR = 0.73) and to non-vascular dementia (RR = 0.64).

To validate the effect of environmental risk factors, this study compared with the model on medication and rehabilitation of stroke patients, as shown in model B. Moreover, this study built an overall multivariate model in which covariates were selected from models A and B. Model C revealed that the environmental risk factors retained the risk effect on transitions. However, two transitions were removed from model C.

### 3.2. Findings in SMP

The predictive model and corresponding result, which includes mean time, standard deviation, and adjusted *R*^2^, are shown in Table 6. The prediction result on overall sojourn time indicated that the transition of diabetes mellitus to ischemic stroke had a sojourn time of 1.96 years. For the transition of stroke to dementia, hemorrhagic stroke to non-vascular dementia had a sojourn time of 1.29 years. The *R*^2^ values in the transition were all greater than 0.8, indicating that the overall prediction model had 80% prediction performance.

**Figure 3:**
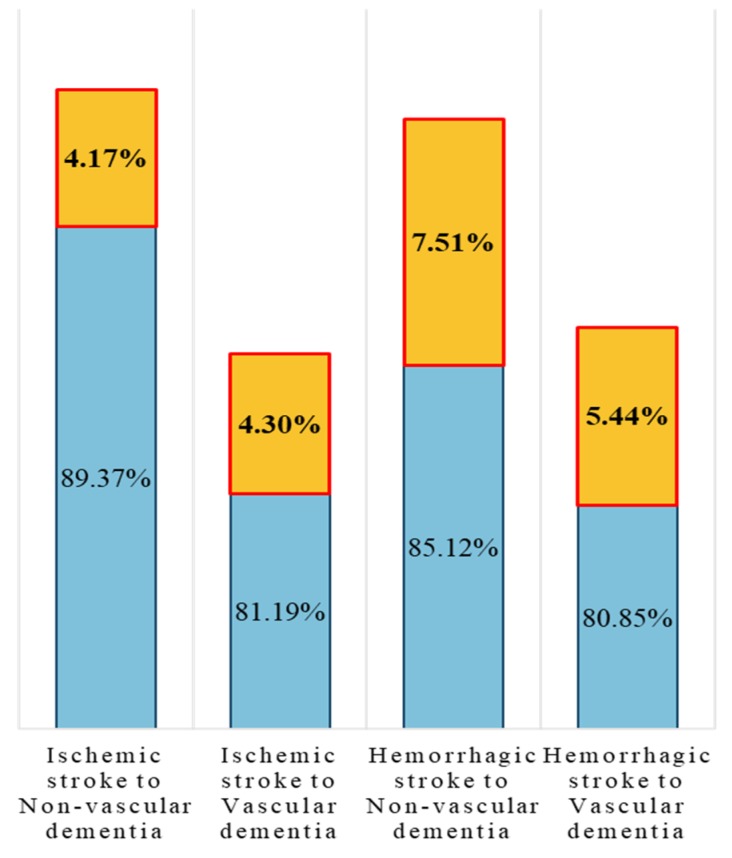
Improvement by the proposed models as compared to only considering medication and rehabilitation.

The results of sojourn time prediction by covariates indicated that different models showed their prediction merit with respect to different chronic diseases. In general, the adjusted *R*^2^ of model A outperformed the others in most transition cases from chronic to both hemorrhagic and ischemic strokes. This result indicated that the environmental risk has high explanatory power in the prediction of transition time. For the transition of stroke to dementia, model C had higher prediction performance than the other models did, except in hemorrhagic stroke to vascular dementia that was preferred by model A. As shown in Figure 3, the transition time prediction was remarkably improved in the proposed model compared with only considering medication and rehabilitation.

Table 7 reveals that the SMP prediction model has high *R*^2^, which indicated that the proposed transition time prediction model has a good explanatory power compared with linear regression, decision tree, random forest, and support vector machine (SVM) [29]. 

Model C considered age, gender, environmental risk factors, and medication and rehabilitation of the stroke patient as covariates. This model indicated that the drugs for lowering blood pressure, blood lipid, and blood sugar decreased the risk of most transitions of chronic disease to stroke and the transition of hypertension to ischemic stroke. In addition, drug interaction data revealed that patients medicated with these three drugs in the same period have low transition risk. Only the blood pressure lowering drug influenced the transition of stroke to dementia (RR = 0.44). For rehabilitation, the results suggested that for people who suffer from ischemic stroke, being active on rehabilitation could reduce their risk of transition to non-vascular dementia (RR = 0.83).

Multivariate analysis results indicated that divorce rate (RR = 3.91), temperature (RR = 1.27, 1.5, 1.72) and air pollution (RR = 1.07, 1.31) have positive signs on chronic disease transfer to stroke. By contrast, the risk of transfer decreases when the unemployment rate shows the negative sign, which indicated high unemployment rate. In the transition of stroke to dementia, the temperature indicated that the risk of transfer to dementia decreased with the temperature (RR = 0.73, 0.64).

After all the environmental factors in multivariate analysis were considered, two transitions were removed from the medication and rehabilitation. These two transitions do not substantially influence the stroke transfer to dementia due to the explanation power of environmental factors.

## 4. Conclusions

This study facilitates accurate prognosis on the transition behavior and time of stroke from chronic diseases to dementias against environmental risks, medication, and rehabilitation factors using a multilayer and multistate SMP model. Results indicated that the covariates incorporated in the study had different effects on sojourn time in stroke transition. Multivariate analysis results revealed that the high divorce rate, high air pollution, and low temperature were high-risk factors that triggered the transition of a chronic disease to stroke. Previous researchers associated cold and hot temperatures with an increased risk of stroke mortality [19,41]. Their study investigated the mortality owing to stroke, whereas our present study confirmed the effect of temperature on the transition of a chronic disease to stroke. Hyperthermia in acute stroke victims caused a poor prognostic parameter on a short-term basis. Roy and Ray [42] focused on body temperature, whereas our present study investigated environmental temperature. Lim et al. [43] addressed that diurnal temperature change over the preceding 24 h is associated with daily stroke incidence. Their study analyzed the short-term effect of environmental temperature, whereas our present study addressed a long-term effect of environmental temperature annually. Age and temperature are high-risk factors for the transition of stroke to dementia. Compared with a covariate model of medication and rehabilitation from a previous study, the proposed environmental factor-based prognosis model had a good explanation power of the risk effects on state transitions. The present study highlighted the high-sensitive population through subgroup analysis. Our result pointed out that the population of males below 65 years old was the most sensitive one against the environmental risk factors. Among all factors, air pollution had the highest influence in the transition of a chronic disease to stroke, and the unemployment rate had the highest influence in the transition of stroke to dementia. 

The constructed SMP model predicts the transition time the stroke patients stay in different states (i.e., from chronic to stroke, and from stroke to dementia). By comparing the adjusted *R*^2^ with other benchmark algorithms (linear regression, decision tree, random forest, and SVM), we found that the proposed prediction model has the highest prediction performance. In addition, we also confirmed that the Weibull distribution has a good flexible fitting ability to deal with medical survival situations. 

Future research may incorporate additional environmental risk factors, such as marital status, socioeconomic status, dietary habits, employment types, and water quality, which may lead to a high prediction on the transition behaviors of stroke. Given the lack of information integration between environmental and medical data, this study connected these data only through the patient’s registered residence. However, integrating these heterogeneous data precisely and individually is still a challenge worthy to be further investigated. Environmental risk factors served as covariates in the proposed model in a binary form due to computational constraints. Future studies may investigate the influence of the numerical types of covariates. The current SMP model may also be extended by adding death state to investigate the survival time of stroke patients.

## Figures and Tables

**Table 1:** ICD-9-CM code definition of all diseases.

Disease Type	Disease Name	ICD-9-CM
Chronic disease	Hypertension	401–405
	Diabetes mellitus	250
	Hyperlipidemia	272
	Chronic lung disease	490–496
	Hyperthyroidism	242
	Chronic kidney disease	585
	Heart failure	428
	Atrial fibrillation	42731
	Sleep apnea	780.51, 780.53, 780.57
	Gout	274
	Peripheral artery disease	437.3, 440, 441, 443.1, 443.2, 443.8, 443.9, 447.1, 447.8, 447.9, 445
Stroke	Hemorrhagic stroke	430, 432
	Ischemic stroke	433, 437
Dementia	Vascular dementia	2904
	Non-vascular dementia	294, 337

**Table 2:** The covariate in the proposed SMP model.

	0 (Base Group)	1
Gender	Female	Male
Age	Otherwise	>65 years old
Divorce rate	Otherwise	High divorce rate
Unemployment rate	Otherwise	High unemployment rate
Elderly living alone rate	Otherwise	High elderly living alone rate
Temperature	Otherwise	Low winter temperature
Air pollution	Otherwise	High rate of PSI > 100

Note: The definition of environmental factors.

**Table 3:** Variable definition.

Environmental Type	Definition
Divorce rate	$\frac{\mathrm{The}\;\mathrm{number}\;\mathrm{of}\;\mathrm{divorced}\;\mathrm{population}\;\mathrm{over}\;15\;\mathrm{years}\;\mathrm{old}}{\;\mathrm{Total}\;\mathrm{population}\;\mathrm{over}\;15\;\mathrm{years}\;\mathrm{old}}\times 100\%$
Unemployment rate	$\frac{\mathrm{The}\;\mathrm{number}\;\mathrm{of}\;\mathrm{unemployed}\;\mathrm{population}}{\mathrm{Labor}\;\mathrm{force}}\times 100\%$
Air pollution	$\frac{\mathrm{Daily}\;\mathrm{PSI}\;>100}{\mathrm{Total}\;\mathrm{monitoring}\;\mathrm{days}\;\mathrm{of}\;\mathrm{PSI}}\times 100\%$
Elderly living alone rate	$\frac{\mathrm{The}\;\mathrm{number}\;\mathrm{of}\;\mathrm{elderly}\;\mathrm{people}\;\mathrm{living}\;\mathrm{alone}}{\;\mathrm{Total}\;\mathrm{population}\;\mathrm{over}\;65\;\mathrm{years}\;\mathrm{old}}\times 100\%$
Temperature	Average of temperature in January, February and December

**Table 4:** The number of states and descriptions in the SMP model.

State	Type	Description
1	Chronic disease	Diagnosed only with hypertension or with one metabolic risk factor after having hypertension
2		Diagnosed only with diabetes mellitus
3		Diagnosed only with hyperlipidemia or with one metabolic risk factor after having hyperlipidemia
4		Diagnosed with more than four metabolic risk factors after having other 8 chronic diseases
5		Diagnosed other than 11 types of chronic disease or without chronic
6	Stroke	Hemorrhagic stroke
7		Ischemic stroke
8	Dementia	Vascular dementia
9		Non-vascular dementia

**Table 5:** The multivariate analysis by different covariates models.

	Model A: Environmental Risk				Model B: Medication and Rehabilitation				Model C: All Factors			
	Transition	Estimation β	Relative Risk	*p*-Value	Transition	Estimation β	Relative Risk	*p*-Value	Transition	Estimation β	Relative Risk	*p*-Value
Gender	β_16_ (1→6)	0.214	1.24	0.0042 *	β_16_ (1→6)	0.183	1.20	0.0143 *	β_16_ (1→6)	0.096	1.10	0.0108
	β_36_ (3→6)	0.493	1.64	0.02 *	β_36_ (3→6)	0.509	1.66	0.0164 *	β_36_ (3→6)	0.490	1.63	0.0206
Age	β_17_ (1→7)	−0.117	0.89	<0.0001 *	β_17_ (1→7)	−0.097	0.91	0.0002 *	β_17_ (1→7)	−0.124	0.88	<0.0001
	β_56_ (5→6)	−2.366	0.09	<0.0001 *	β_56_ (5→6)	−2.382	0.09	<0.0001 *	β_56_ (5→6)	−2.374	0.09	<0.0001
	β_57_ (5→7)	−2.538	0.08	<0.0001 *	β_57_ (5→7)	−2.538	0.08	<0.0001 *	β_57_ (5→7)	−2.541	0.08	<0.0001
	β_69_ (6→9)	−0.394	0.67	0.0259 *	β_69_ (6→9)	−0.394	0.67	0.0259 *	β_69_ (6→9)	−0.393	0.68	0.0259
	β_79_ (7→9)	−0.372	0.69	0.0008 *	β_79_ (7→9)	−0.389	0.68	0.0005 *	β_79_ (7→9)	−0.366	0.69	0.001
Divorce rate	β_26_ (2→6)	1.364	3.91	0.0122 *					β_26_ (2→6)	1.364	3.91	0.0123
Unemployment rate	β_16_ (1→6)	−0.434	0.65	0.0004 *					β_16_ (1→6)	−0.447	0.64	0.0003
	β_17_ (1→7)	−0.369	0.69	<0.0001 *					β_17_ (1→7)	−0.365	0.69	<0.0001
	β_36_ (3→6)	−0.673	0.51	0.0379 *					β_36_ (3→6)	−0.673	0.51	0.0377
	β_37_ (3→7)	−0.486	0.62	<0.0001 *					β_37_ (3→7)	−0.493	0.61	<0.0001
	β_47_ (4→7)	−0.904	0.40	<0.0001 *					β_47_ (4→7)	−0.915	0.40	<0.0001
Temperature	β_16_ (1→6)	0.237	1.27	0.0088 *					β_16_ (1→6)	0.222	1.25	0.0142
	β_17_ (1→7)	0.408	1.50	<0.0001 *					β_17_ (1→7)	0.397	1.49	<0.0001
	β_27_ (2→7)	0.540	1.72	<0.0001 *					β_27_ (2→7)	0.625	1.87	<0.0001
	β_37_ (3→7)	0.237	1.27	0.0015 *					β_37_ (3→7)	0.227	1.25	0.0023
	β_78_ (7→8)	−0.316	0.73	0.0411 *					β_78_ (7→8)	−0.316	0.73	0.0409
	β_79_ (7→9)	−0.444	0.64	<0.0001 *					β_79_ (7→9)	−0.446	0.64	<0.0001
Air pollution	β_17_ (1→7)	0.071	1.07	0.0057 *					β_17_ (1→7)	0.079	1.08	0.0035
	β_47_ (4→7)	0.272	1.31	0.0059 *					β_47_ (4→7)	0.260	1.30	0.0085
Blood pressure lowing drug					β_16_ (1→6)	−0.275	0.76	0.0042 *	β_16_ (1→6)	−0.318	0.73	0.0009
					β_17_ (1→7)	−0.458	0.63	<0.0001 *	β_17_ (1→7)	−0.469	0.63	<0.0001
					β_37_ (3→7)	−0.218	0.80	0.0006 *	β_37_ (3→7)	−0.215	0.81	0.0008
					β_47_ (4→7)	−0.331	0.72	0.0309 *	β_47_ (4→7)	−0.351	0.70	0.0222
					β_68_ (6→8)	−0.822	0.44	<0.0001 *	-	-	-	-
Blood lipid lowing drug					β_17_ (1→7)	−0.261	0.77	<0.0001 *	β_17_ (1→7)	−0.250	0.78	<0.0001
Glycemic lowing drug					β_17_ (1→7)	−0.132	0.88	0.0168 *	β_17_ (1→7)	−0.150	0.89	0.0068
					β_27_ (2→7)	−0.254	0.78	0.0119 *	β_27_ (2→7)	−0.349	0.71	0.0007
Thrombus prevention drug					β_16_ (1→6)	−0.237	0.79	0.0053 *	β_16_ (1→6)	0.096	1.10	0.0108
Rehabilitation					β_79_ (7→9)	−0.187	0.83	0.0243 *	-	-	-	-

**Table 6:** Sojourn time prediction.

**Overall Sojourn Time Prediction**										
Chronic to Stroke					Hemorrhagic Stroke			Ischemic Stroke		
					Mean (Year)	SD	R^2^ *	Mean	SD	R^2^
State 1: Hypertension					2.94	2.94	92.3%	2.72	2.89	87.0%
State 2: Diabetes mellitus					2.48	2.50	90.2%	1.96	2.36	86.1%
State 3: Hyperlipidemia					2.93	2.94	95.5%	2.63	2.80	92.9%
State 4: Other 8 chronic diseases					4.67	3.74	92.3%	4.77	3.71	97.5%
State 5: Other than above 11 types of chronic disease or without chronic					25.53	20.24	90.3%	29.03	22.65	91.3%
Stroke to Dementia					State 8: Vascular dementia			State 9: Non-vascular dementia		
					Mean	SD	R^2^	Mean	SD	R^2^
State 6: Hemorrhagic stroke					1.94	2.23	77.3%	1.29	2.02	80.0%
State 7: Ischemic stroke					2.61	2.85	83.4%	2.71	2.79	88.6%
**Sojourn Time Prediction by Different Covariates Models**										
Covariate Transition		A: Environmental Risk			B: Medication and Rehabilitation			C: All Factors		
Hemorrhagic stroke		Mean (Year)	SD	R^2^	Mean (Year)	SD	R^2^	Mean (Year)	SD	R^2^
	β_16_ (1→6)	2.79	2.86	95.34%	3.19	3.06	93.11%	3.12	3.05	94.07%
	β_26_ (2→6)	2.36	2.45	90.92%	2.37	2.43	91.81%	2.24	2.36	89.23%
	β_36_ (3→6)	2.80	2.88	95.06%	2.68	2.79	94.28%	2.97	3.01	93.61%
	β_46_ (4→6)	7.03	3.56	91.73%	6.83	3.68	91.49%	7.04	3.58	90.82%
	β_56_ (5→6)	30.22	22.70	90.33%	29.82	22.62	90.35%	30.19	22.68	92.31%
Ischemic stroke		Mean (yr)	SD	R^2^	Mean (yr)	SD	R^2^	Mean (yr)	SD	R^2^
	β_17_ (1→7)	2.71	2.90	92.63%	3.15	3.10	87.83%	3.11	3.09	92.30%
	β_27_ (2→7)	1.86	2.30	88.28%	1.97	2.37	87.47%	1.93	2.35	86.08%
	β_37_ (3→7)	2.55	2.76	93.98%	2.65	2.81	93.26%	2.64	2.82	93.06%
	β_47_ (4→7)	5.03	3.88	96.04%	5.56	3.93	97.07%	5.69	4.02	96.34%
	β_57_ (5→7)	37.15	25.55	91.22%	36.97	25.51	91.23%	37.15	25.55	91.57%
Vascular dementia		Mean (yr)	SD	R^2^	Mean (yr)	SD	R^2^	Mean (yr)	SD	R^2^
	β_68_ (6→8)	1.68	2.15	91.79%	2.25	2.36	80.85%	2.39	2.38	86.29%
	β_69_ (6→9)	3.01	3.05	87.23%	3.10	3.07	85.12%	3.16	3.07	92.63%
Non-vascular dementia		Mean (yr)	SD	R^2^	Mean (yr)	SD	R^2^	Mean (yr)	SD	R^2^
	β_78_ (7→8)	1.44	2.13	78.96%	1.42	2.12	81.19%	1.44	2.13	85.49%
	β_79_ (7→9)	3.07	2.99	90.07%	3.11	3.00	89.37%	3.17	3.03	93.54%

* Note: Adjusted *R*^2^ = $1-\left(1-R^{2}\right)\frac{n-1}{n-p-1}$, where *p* is the number of regressors, and *n* is the sample size.

**Table 7:** *R*^2^ of SMP comparison with benchmark models.

Hemorrhagic Stroke	Linear Regression	Decision Tree	Random Forest	SVM	SMP
Environmental risk	88.52%	91.35%	88.84%	91.93%	92.66%
Medication and Rehabilitation	88.41%	91.87%	88.49%	90.22%	92.22%
All factors	88.50%	92.00%	91.60%	90.19%	92.01%
Ischemic stroke					
Environmental risk	85.63%	88.57%	85.09%	89.40%	92.42%
Medication and Rehabilitation	85.36%	90.06%	85.81%	90.00%	91.38%
All factors	85.60%	90.35%	91.57%	90.56%	91.88%
Vascular dementia	Linear Regression	Decision Tree	Random Forest	SVM	SMP Model
Environmental risk	75.74%	79.61%	81.18%	73.28%	89.51%
Medication and Rehabilitation	77.46%	82.61%	82.88%	75.54%	83.00%
All factors	78.76%	85.27%	86.91%	78.52%	89.46%
Non-vascular dementia					
Environmental risk	75.80%	76.44%	77.70%	73.60%	84.52%
Medication and Rehabilitation	77.87%	81.11%	81.29%	76.77%	85.28%
All factors	75.66%	82.96%	84.45%	81.67%	89.52%
